# Use of 5‐Thio‐L‐Fucose to modulate binding affinity of therapeutic proteins

**DOI:** 10.1002/bit.27695

**Published:** 2021-02-19

**Authors:** Martina Zimmermann, Melanie Nguyen, Christian M. Schultheiss, Harald Kolmar, Aline Zimmer

**Affiliations:** ^1^ Life Science, Upstream R&D Merck KGaA Darmstadt Germany; ^2^ Institute for Organic Chemistry and Biochemistry Technische Universität Darmstadt Darmstadt Germany

**Keywords:** antibody‐dependent cellular cytotoxicity, CHO cell culture, FcγRIIIa, fucosylation, fucose analogue

## Abstract

The reduction of antibody core‐fucosylation is known to enhance antibody‐dependent cellular cytotoxicity (ADCC). In this study, 5‐Thio‐l‐Fucose (ThioFuc) was investigated as a media and feed supplement for modulating the fucosylation profile of therapeutic proteins and, thereby, improving the resulting effector functions. Glycan analysis of five different therapeutic proteins produced by a diverse set of Chinese hamster ovary cell lines demonstrated a clone dependent impact of ThioFuc treatment. Using rituximab as a model, an efficient dose‐ and time‐dependent reduction of core‐fucosylation up to a minimum of 5% were obtained by ThioFuc. Besides a concomitant increase in the afucosylation level up to 48%, data also revealed up to 47% incorporation of ThioFuc in place of core‐fucosylation. In accordance with the glycan data, antibodies produced in the presence of ThioFuc revealed an enhanced FcγRIIIa binding up to 7.7‐fold. Furthermore, modified antibodies subjected to a cell‐based ADCC reporter bioassay proved to exert both a 1.5‐fold enhanced ADCC efficacy and 2.6‐fold enhancement in potency in comparison to their native counterparts—both of which contribute to an improvement in the ADCC activity. In conclusion, ThioFuc is a potent fucose derivative with potential applications in drug development processes.

## Graphical abstract

Discovery of 5‐Thio‐L‐fucose, a potent cell culture media supplement capable to reduce antibody core‐fucosylation. Modified antibodies revealed an enhanced FcyRIIIa binding and an improved ADCC activity known to improve drug potency.

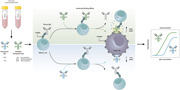

## 1 INTRODUCTION

Recombinant proteins, including therapeutic antibodies, are commonly produced in mammalian cell lines such as Chinese hamster ovary (CHO) cells. The bifunctional structure of antibodies enables target antigen binding and simultaneous binding of effector cell receptors via the crystallizable fragment (Fc). The responsible receptors are designated as Fc gamma receptors (FcγR), due to their specificity to the gamma heavy chain of immunoglobulin G (IgG). FcγRI (CD64) is classified as high‐affinity receptor, whereas FcγRII (CD32) and FcγRIII (CD16) are known as low‐affinity receptors (Bournazos et al., 2009). FcγR trigger different immune responses depending on the cytoplasmic domains. Common domains are immunoreceptor tyrosine‐based activation motifs (ITAMs) for activating receptors (FcγRI, FcγRIIa, FcγRIIc, FcγRIIIa, and FcγRIIIb) and immunoreceptor tyrosine‐based inhibition motifs (ITIMs) for inhibitory receptors (FcγRIIb; Lu & Sun, 2015). Both downstream signaling pathways of these receptors are required for the control of cellular activation toward the antibody driven immune response (Long, 1999; Ravetch & Bolland, 2001).

Antibodies belonging to the subclass IgG1, as common therapeutic monoclonal antibodies (mAbs), are reported to bind all classes of receptors with increasing affinities for FcγRIIb/c, FcγRIIIb, FcγRIIIa, and FcγRIIa and the highest affinity for FcγRI (Bruhns et al., 2009). The low‐affinity receptor FcγRIIIa is particularly important for the clinical efficacy of known therapeutic antibodies such as rituximab (Rituxan®; Cartron et al., 2002; Dall'Ozzo et al., 2004), since its engagement induces an ADCC response. FcγRIIIa is expressed by natural killer (NK) cells, macrophages, monocytes, and several T‐cells (Bournazos et al., 2009), whereby the former is mainly responsible for ADCC activity through the release of cytotoxic granules and subsequent lysis of target cells (Seidel et al., 2013; Zahavi et al., 2018). Clinical studies revealed that antibody potency is influenced by the FcγRIIIa phenotype in patients caused by single‐nucleotide polymorphism (Bournazos et al., 2009; Mellor et al., 2013). Phenotypes and related receptor activity are defined by amino acids at positions 48 and 158 (Koene et al., 1997). Position 158 can be either phenylalanine (F) or valine (V), with F having a bulkier structure. This modification was suggested to lead to steric hindrance and reduce antibody affinity, which is key for the therapeutic potency (Treon et al., 2005). According to clinical studies with rituximab, patients with homozygote V/V or heterozygote V/F phenotypes showed an enhanced response to the treatment compared to a homozygote F/F (Cartron et al., 2002). Although patients with at least one V allele were reported to exhibit increased receptor expression in NK cells, the clinical response was only related to the high affinity (Congy‐Jolivet et al., 2008; Hatjiharissi et al., 2007). Nevertheless, genotypes did not always correlate with the antitumor activity, indicating that the polymorphism contributes but is not decisive for the clinical outcome (Mellor et al., 2013).

Besides FcγR polymorphisms, receptor affinity and, thereby, the biological activity of an antibody depends on the glycosylation of both antibody and receptor (Cambay et al., 2019; Dashivets et al., 2015; Hayes et al., 2014, 2017). For FcγRIIIa, five N‐linked glycosylation sites are known, whereby two glycans at asparagine 45 (Asn‐45) and Asn‐162 were reported to highly affect the binding affinity of antibodies. The glycan at position 45 was reported to hinder a high‐binding affinity, while glycosylation N‐162 was required (Mizushima et al., 2011; Shibata‐Koyama et al., 2009). Since the glycosylation pattern of receptors in patients is difficult to modulate, antibody glycosylation has become the focus of attention in drug development.

IgG1 commonly have a heterogeneous glycosylation pattern at position 297 (Asn‐297) on each heavy chain in the Fc‐part. The content of galactose (Kumpel et al., 1995; Thomann et al., 2015), bisecting N‐acetylglucosamine (GlcNAc), and especially core‐fucose has been reported to affect ADCC. A 50‐fold enhanced affinity to the receptor, leading to 100‐fold enhanced ADCC, was detected by treating non‐Hodgkin lymphoma cells with anti‐CD20 antibodies containing bisected and afucosylated glycans compared to highly fucosylated anti‐CD20 antibodies (Umana et al., 2006). Furthermore, in vitro detected enhanced ADCC was correlated with improved potency of afucosylated antibodies in vivo (Niwa et al., 2004). Further in vivo studies of afucosylated anti‐EGFR and anti‐CS1 antibodies presented enhanced ADCC and antitumor activity when compared to their fucosylated counterparts (Gerdes et al., 2013; Gomathinayagam et al., 2015). Overall, the fucosylation level is often described as the predominant determinant for enhanced ADCC activity (Thomann et al., 2016).

Several explanations are reported in the literature for the potency boost provided by afucosylated antibodies. Crystal structure analysis of the antibody‐receptor complex indicates two possible tyrosine‐296 orientations of the aromatic ring within IgG, leading to a high‐ and low‐affinity binding mode. Afucosylated antibodies were suggested to favor the tyrosine conformation, which leads to the high‐affinity binding mode. In contrast, steric hindrance through core‐fucosylated IgG favors the alternative conformation and thus the low‐affinity binding (Mizushima et al., 2011). Additionally, carbohydrate‐carbohydrate interactions between receptor and afucosylated Fc‐glycans were reported to enhance the antibody binding by stabilizing the complex (Ferrara et al., 2011; Lu & Sun, 2015).

Since modulation of Fc fucosylation is a powerful strategy to enhance the potency of therapeutic antibodies, several fucose analogues or acetylated derivatives have been reported with respect to their ability to reduce core‐fucosylation by acting as competitive inhibitors of fucosyltransferase 8 (FUT8) and triggering substrate feedback inhibition (Okeley, Alley, et al., 2013; Zimmermann et al., 2019). Some of these have also been applied in cell culture to successfully reduce core‐fucosylation: 2‐deoxy‐2‐fluorofucose (2F‐Fucose; Rillahan et al., 2012; Zhou et al., 2017; Zimmermann et al., 2019), 5‐Alkynyl‐Fucose (Kizuka et al., 2017; Okeley, Toki, et al., 2013; Zimmermann et al., 2019), 6,6,6‐trifluorofucose (McKenzie et al., 2018), and l‐fucose phosphonate (Allen et al., 2014, 2016). These publications focus on removal, changed orientation, or substitution of hydroxyl groups with various functional groups to inhibit enzymes in either the de novo or the salvage pathway required for core‐fucosylation.

In this study, a novel application of 5‐Thio‐l‐Fucose (ThioFuc) was demonstrated, namely the supplementation during cell cultivation for producing antibodies with enhanced ADCC activity. To study the impact of this treatment, rituximab, a monoclonal IgG1 targeting the CD20 antigen, was utilized as a model antibody. Obtained data confirmed that ThioFuc supplementation led to an enhanced antibody‐receptor binding and consequently enhanced ADCC activity.

## 2 MATERIALS AND METHODS

### 2.1 Materials

Ac2F‐Fuc was purchased from Merck (344827) and ThioFuc as well as AcThioFuc were synthesized by WuXi AppTec. The identity was confirmed via nuclear magnetic resonance.

### 2.2 Fed‐batch cultivation

Five different CHO clones expressing four different mAbs including rituximab and a fusion protein were tested. Cells were cultivated in spin tubes between 14 and 20 days at 37°C, 5% CO_2_, and 80% humidity. Two independent cell culture experiments were performed using clone 1 producing rituximab (mAb1), with starting cell densities of either 2 × 10^5^ or 3 × 10^5^ cells/ml in 30 mL Ex‐Cell® Advanced Media (Merck 24366C) with a pH of 7.2 ± 0.1 (Ex‐Cell® Advanced Platform) and an agitation rate of 320 rpm. Depending on the experiment, cells were fed on Day 3 with 3% volume (v/v) of Ex‐Cell® Advanced Feed (Merck 24368C) and on Days 5, 7, 10, and 12 with 6% (v/v) of the feed, or with 5% (v/v) on Days 3, 5, 7, 10, and 12. Data of both experiments were merged, as only minor differences were detected. For clones 2 and 3 producing mAb2 and mAb3 each, a starting cell density of 3 × 10^5^ cells/ml in 30 mL Cellvento® 4CHO media COMP (Merck 103795) with a pH of 7.0 ± 0.1 was used (Cellvento® Platform). Cells were cultivated at an agitation rate of 320 rpm and were fed on Days 3, 5, 10, 12, and 14 with 3% volume (v/v) of Cellvento® 4Feed (Merck 103796) and on Day 7 with 6% (v/v) of the feed. Clones 4 and 5 producing mAb4 and a fusion protein were inoculated at the same cell density in 30 ml Ex‐Cell® Advanced Media, while agitation was set to 230 rpm. Clones 4 and 5 were fed with 5% Ex‐Cell® Advanced Feed (v/v) on Days 3, 5, 7, 10, 12, 14, and 17. Feeds were supplemented with either ThioFuc, AcThioFuc, Ac2F‐Fuc as positive control or the respective amount of DMSO (1.17%; Merck 102931) used as solvent for both acetylated fucose analogues. As all treatments were part of the feed solution, first supplementation was on Day 3 and applied concentration accumulated over time due to several feed additions. The pH of all Cellvento® Feeds was adjusted to 7.0 ± 0.1 and Ex‐Cell® Feeds was adjusted to 8.5 ± 0.1. The glucose level was maintained above 4 g/L by adding a specific amount of a 400 g/L glucose stock solution on demand to up to 6 g/L during the week and up to 13 g/L over the weekend. Glucose and titer were detected in the supernatant with the bioprocess analyzer CEDEX Bio HT (Roche) based on spectrophotometric and turbidometric methods. The viable cell density (VCD) and viability were evaluated with a Vi‐CELL™ XR 2.04 cell counter (Beckman Coulter).

### 2.3 Perfusion cultivation

Two 4‐L perfusion bioreactor (Applikon Biotechnology) cultures were compared using Ex‐Cell® Advanced HD Perfusion Medium (Merck 24370C) with and without supplementation of 25 µM ThioFuc. Both cultures were inoculated at 0.5 × 10^6^ viable cells/ml and expanded 3 days in batch mode at 37°C, pH 7.0 ± 0.05 and dissolved oxygen of 40 ± 5%. On Day 3, bioreactors were perfused with the same medium with one vessel volume exchange per day (1 vvd). Achieving 35 × 10^6^ viable cells/ml, both bioreactors were further controlled for 14 days in steady‐state using a capacitance probe (Aber Instruments Ltd) to maintain a constant VCD. Bleed and harvest flow were automatically adapted (SecureCell AG) to each other to achieve a constant perfusion rate of 1 vvd. Samples of the last 7 days in steady‐state were analyzed to compare the effect of supplementation.

### 2.4 Antibody purification and analysis of the glycosylation pattern

The antibody was purified from the cell culture supernatant by protein A PhyTips® with the semi‐automated system Pure Speed (PhyNexus Inc). The relative quantitative analysis of the N‐linked glycans was performed after glycan cleavage from the antibody and derivatization using the GlycoWorks™ RapiFluor‐MS™ *N*‐Glycan Kit (Waters 176003713) and with ultra‐performance liquid chromatography coupled to a mass spectrometer (UPLC‐MS). UPLC analysis was performed according to the manufacturer's protocol using an ACQUITY UPLC Glycan BEH Amide Column (300 Å, 1.7 µm, 2.1 × 150 mm^2^) coupled to an ACQUITY UPLC® FLR Detector (Ex: 265 nm, Em: 425 nm) as described in previous studies (Zimmermann et al., 2019). Identification of the glycan structures was performed using a dextran calibration ladder (Waters 186007982) to detect the specific retention time and MS spectra by calculating the mass to charge ratio with an electrospray ionization (ESI) source in positive mode (Synapt G1 HDMS, Waters). The resulting retention time in combination with the obtained mass was used to identify the distinct glycan structures, when the signal intensity was over the detection limit of 0.2%. Incorporation of ThioFuc was detected through a mass shift in the MS spectra of +15.98 Da. The highest detected mass error within all experiments was ±13 ppm. For quantification of the glycan contents, the relative peak area was determined using a fluorescence detector and integration of extracted‐ion chromatogram was used to separate overlapping peaks. Thus, the percentage of each glycoform was quantified by calculating the ratio of the peak area of each glycan to the sum of all peak areas.

### 2.5 Detection of binding affinity via surface plasmon resonance

The binding of samples after ThioFuc treatment was investigated by surface plasmon resonance (SPR) technology using a Biacore™ T200 system (Cytiva). The analysis temperature was set to 25°C and sample compartment temperature to 15°C. Using the Amine Coupling Kit (Cytiva BR100050) and the His Capture Kit (Cytiva 28995056) from Cytiva, a series S Sensor Chip CM5 (Cytiva 29149603) was used to couple anti‐His antibody according to the manufacturer's instructions in the active and the reference flow cell. Immobilization levels were in a range of 12,373 ± 587 resonance units (RU; 1 RU ≈ 1 pg/mm^2^) for all experiments. The anti‐His antibody was further used to capture His‐tagged FcγR from AcroBiosystems. 0.022 µg/ml FcγRI (FCA‐H52H2), 0.75 µg/ml FcγRIIb (CDB‐H5228), 0.033 µg/ml FcγRIIIa F176 (CDA‐H82E8), and 0.033 µg/ml FcγRIIIa V176 (CD8‐H52H4) were injected in the active flow cell for 60 s at a flow rate of 10 µl/min, resulting in capture levels of 12 RU, 404 RU, 8 RU, and 16 RU, respectively. Subsequently, rituximab was injected for 150 s at a flow rate of 30 µl/min with five increasing concentrations over both, the reference and active flow cells. For all steps, the HBS‐EP+ Buffer (Cytiva BR100669) was used. Binding was recorded continuously, while unspecific binding and injection artefacts were subtracted automatically. Single‐cycle kinetics were used with a dissociation time of 600 s at a flow rate of 30 µl/min. Data collection was performed at a rate of 10 Hz. Following each experiment, flow cells were regenerated for 30 s with 10 mM glycine‐HCl pH 1.5 (Cytiva BR100354) according to the kit instructions. Data of three technical and two biological replicates were fitted with a 1:1 binding model. The global dissociation constant *K*_D_ was determined from the ratio of the kinetic rate constants for dissociation (*k*_d_) and association (*k*_a_). For FcγRIIb, the steady‐state model was applied, setting the report points directly 15 s after injection start.

### 2.6 ADCC assay

The ADCC activity of the antibody of interest and possibly changes upon cell treatment with fucose analogues were measured using the commercially available ADCC Reporter Bioassay Kit according to the manufacturer's instructions (Promega, G7015). The CD20^+^ target cells (Raji cells) and effector cells (engineered Jurkat cells) were resuspended in RPMI 1640 medium including 4% low IgG serum (ADCC assay buffer). The anti‐CD20 antibody (rituximab) or the biosimilar truxima® were applied in a threefold dilution series starting at 1 μg/ml, and an effector‐target cell ratio (E:T) of 6:1. After preparation, the plate was incubated at 37°C, 5% CO_2,_ and 80% humidity for 6 h. By adding 75 μl of Bio‐Glo™ Luciferase Assay Reagent, the ADCC response was quantified via luminescence.

## 3 RESULTS

**Figure 1:**
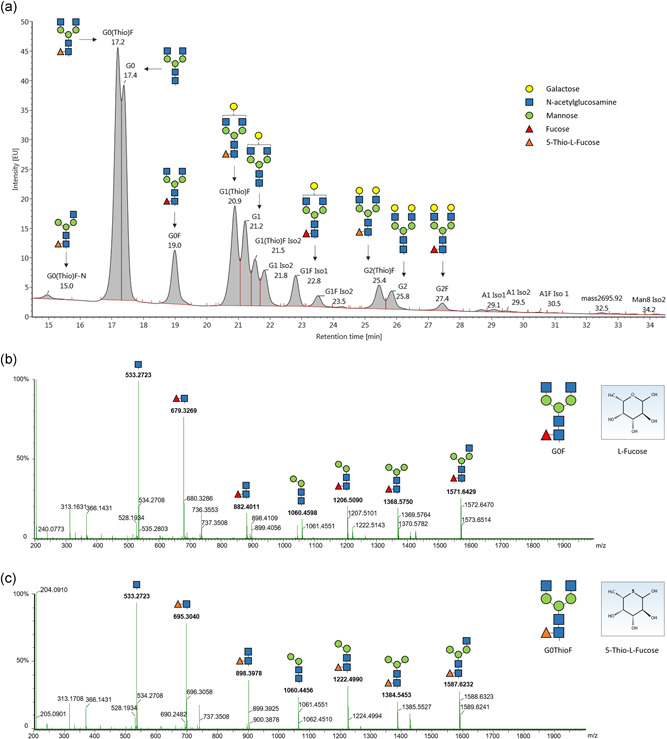
LC‐MS analysis of released glycans suggests ThioFuc integration in place of core‐fucosylation in rituximab. (a) Fluorescence signal obtained after separation of the released, labeled glycans using UPLC, showing a changed retention time of the peaks corresponding to thiofucosylated glycans compared to fucosylated glycans. Comparison of the (b) fucosylated G0F and (c) thiofucosylated G0ThioF fragmentation pattern. LC‐MS, liquid chromatography‐mass spectrometry; UPLC, ultra‐performance liquid chromatography [Color figure can be viewed at wileyonlinelibrary.com]

The reduction of core‐fucosylation in the Fc‐part of antibodies is known to induce enhanced ADCC and cell culture media/feed supplementation is an elegant strategy to achieve this without cell line engineering. Many studies tested fucose derivatives with various hydroxyl group replacements, whereas in our study the ring oxygen in fucose was exchanged with sulfur leading to ThioFuc (Figure 1).

### 3.1 5‐Thio‐l‐Fucose supplementation in cell culture leads to IgG bearing thiofucosylated glycans

A common fed‐batch process was performed to determine the effect of 200 µM ThioFuc as feed supplement on the glycosylation profile of an IgG1. Due to the high structural similarity of ThioFuc to the native l‐Fucose, incorporation of ThioFuc was thought to be likely. Following harvest of rituximab on Day 12, the antibody was purified from the supernatant and the glycosylation profile was investigated via UPLC‐MS/MS. In the chromatogram of rituximab produced in presence of ThioFuc, several peaks were identified as thiofucosylated species via MS/MS (Figure 1a). The exchange of the ring oxygen in fucose (164.0684 g/mol) with sulfur in the ThioFuc derivative (180.0455 g/mol) causes a mass shift of about +16 Da, allowing clear discrimination of both sugars. The comparison of the glycan fragments indicates that ThioFuc is attached to the core‐GlcNAc, since the fragmentation pattern of fucosylated glycans like G0F (Figure 1b) is similar to the respective glycans containing ThioFuc (Figure 1c). Thiofucosylation at a different position of the glycan antennae leading to further glycan species was not observed.

### 3.2 Dose‐dependent impact of ThioFuc on cell performance and the glycosylation profile

**Figure 2:**
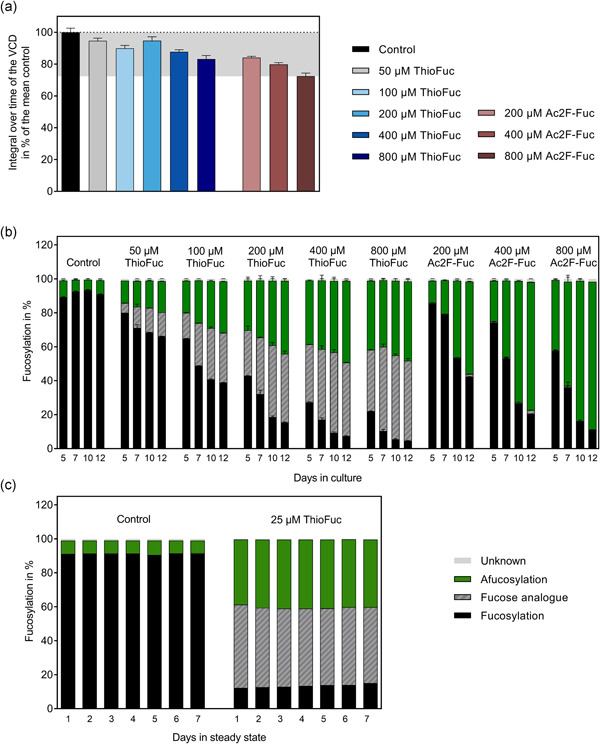
The impact of ThioFuc on CHO cells producing rituximab. Suspension CHO cells were seeded at 3 × 10^5^ cells/ml, incubated at 37°C, 5% CO_2_, 80% humidity, and agitated at 320 rpm for fed‐batch experiments. Feed with increasing ThioFuc or Ac2F‐Fuc concentrations was added on Days 3, 5, 7, 10, and 12 (5%; v/v). (a) Integral over time of the VCD. (b) Dose‐dependent fucosylation profile over time in % of all N‐glycans. (c) Fucosylation profile of rituximab produced during steady state at 1 vvd and 3.5 × 10^7^ cells/ml with and without 25 µM ThioFuc in the media. All presented N‐glycans were identified by UPLC‐MS analysis and thiofucosylated glycans were detected via a 16 Da mass shift. Glycan data are visualized as mean values ± standard error of the mean (*SEM*; *n* = 1 for perfusion, *n* = 4 for 200, 400, and 800 µM ThioFuc, and *n* = 2 for all the other treatments). CHO, Chinese hamster ovary; UPLC‐MS, ultra‐performance liquid chromatography‐mass spectrometry [Color figure can be viewed at wileyonlinelibrary.com]

To determine the effect of ThioFuc on cell performance and the glycosylation profile, increasing concentrations from 50 to 800 µM ThioFuc were applied in fed‐batch experiments producing rituximab (mAb1). 2F‐Peracetylfucose (Ac2F‐Fuc), a fucose analogue known to reduce core‐fucosylation, was used as positive control (Rillahan et al., 2012). The VCD measured in fed‐batch experiments is visualized as mean integral over time and normalized to the respective untreated control condition. Compared to the control, supplementation of ThioFuc showed reduced VCD at increasing concentrations, with a maximum reduction of 17% upon application of 800 µM ThioFuc (Figure 2a). However, cell viability and titer contributing to the cell performance were not affected, irrespective of the applied ThioFuc concentration (Figure S1a,b).

As ThioFuc supplementation during the fed‐batch was intended to modify the fucosylation level, the glycosylation was investigated repetitively between Days 5 and 12 at increasing ThioFuc concentrations. The control condition without supplementation exhibited inherently high fucosylation levels in a range from 89% to 93% between Days 5 and 12 (Figure 2b). As opposed to the control, supplementing increasing concentrations of ThioFuc into the cell culture feed reduced fucosylation over time leading to 66%, 39%, 15%, 7%, and 5% fucosylation on Day 12 upon feed supplementation with 50, 100, 200, 400, and 800 µM ThioFuc, respectively.

In‐depth investigation showed that fucosylation of the control on Day 12 (91%) was mainly comprised of the glycans G0F (52%), G1F (33%), and G2F (4%) as main fucosylated glycans (Table S1). The addition of 400 µM ThioFuc led to a reduction of the total fucosylation to 7% on Day 12 through simultaneous reduction of G0F, G1F, and G2F glycan structures to 4%, 3%, and <1%. In contrast, afucosylated species G0, G1, and G2 were increased from 4%, 2%, and <1% in the control to 30%, 13%, and 1%, respectively by treatment with 400 µM ThioFuc. In addition, 400 µM ThioFuc treatment gave rise to 30% G0ThioF, 12% G1ThioF, and 2% G2ThioF, leading to a total of 43% thiofucosylation (Figure 2b). Overall, application of increasing ThioFuc concentrations induced a dose‐dependent increased thiofucosylation from 14% by 50 µM ThioFuc to 47% by 800 µM ThioFuc and afucosylation level was similarly increased from 18% to 47%, respectively. Besides fucosylation, ThioFuc treatment resulted in a dose‐dependent reduced galactosylation from 40% in the control to 30% through 800 µM ThioFuc on Day 12, whereas mannosylation and sialylation remained unchanged (Figure S1c). These data demonstrate that ThioFuc feed supplementation causes a dose‐dependent increase of antibodies that are afucosylated and thiofucosylated.

Comparing the fucosylation profile of 200 µM ThioFuc and 200 µM Ac2F‐Fuc treatments over time, 29.1% afucosylation was observed for ThioFuc compared to 13.0% for Ac2F‐Fuc on Day 5. These data indicate a faster response with higher afucosylation by ThioFuc, which might be beneficial for batch cultivation as well as perfusion processes.

To assess whether ThioFuc can be applied in other manufacturing processes, rituximab was produced in a 4‐L perfusion bioreactor, while media with or without 25 µM ThioFuc were exchanged daily. Glycosylation analysis during steady‐state growth showed a constant fucosylation level of 91% in the control, which was reduced to 13% in presence of 25 µM ThioFuc (Figure 2c). In contrast, afucosylation in the control (8%) was increased to 40% by 25 µM ThioFuc, whereby 46% thiofucosylation was observed. This indicates that ThioFuc can be applied in both fed‐batch and perfusion processes resulting in modified antibody glycosylation patterns.

### 3.3 Thiofucosylation and afucosylation levels are clone dependent

**Figure 3:**
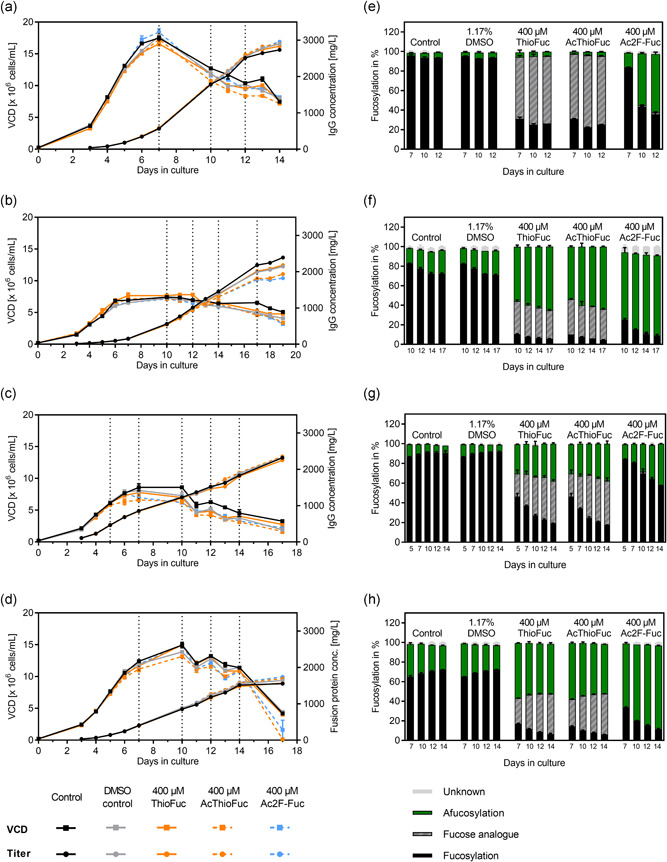
Impact of ThioFuc on cell performance during a fed‐batch experiment using four CHO clones and the respective fucosylation profile on several days. Two different platforms with the respective media, feed and feeding strategy were used. VCD and titer of (a) clone 2 and (b) clone 3 were cultivated with the Cellvento® platform, whereby (c) clone 4 and (d) clone 5 were cultivated with the Ex‐Cell® Advanced platform (*n* = 4). The days for which the fucosylation level was investigated are indicated as a dotted line. The fucosylation level of clones 2, 3, 4, and 5 producing (e) mAb2, (f) mAb3, (g) mAb4, and (h) a fusion protein, respectively are presented as mean values ± *SEM* of two biological replicates. CHO, Chinese hamster ovary; VCD, viable cell density [Color figure can be viewed at wileyonlinelibrary.com]

The applicability of ThioFuc for different drug manufacturing processes was assessed by comparing four different clones producing three different mAbs and an Fc‐fusion protein in presence of 400 µM ThioFuc. To address potential ThioFuc uptake limitation in these clones, equimolar concentrations of acetylated ThioFuc (AcThioFuc) and Ac2F‐Fuc were tested as well. Both acetylated fucose analogues were dissolved in dimethyl sulfoxide (DMSO), leading to 1.17% DMSO in the feed. Solvent related effects were assessed using a DMSO control. The cell performance and glycosylation profile were investigated for each clone using two cultivation platforms. The Cellvento® platform was used to cultivate clone 2—a fast‐growing and high producer clone (mAb2) and clone 3 with a rather slow growth but long stationary phase producing mAb3 (Figure 3a,b). The Ex‐Cell® Advanced platform was used to cultivate another slow‐growing clone 4 (mAb4) and a fast‐growing clone 5 producing a fusion protein (Figure 3c,d). Within four biological replicates, ThioFuc treatment with or without acetylation showed no significant impact on cellular performance (VCD and titer) of these CHO cell lines when compared to the controls. The Fc‐glycosylation profile of all therapeutic proteins was investigated on several days depending on titer (>400 mg/L) and cell viability (>60%).

Treatment with either ThioFuc (irrespective of acetylation) or the positive control showed drastic changes regarding the fucosylation levels for all therapeutic proteins on all days, whereby DMSO was not critical. For clone 2, ThioFuc seemed to be a good substrate of the fucosyltransferase, since 69% of mAb2 was thiofucosylated (25% fucosylated) as opposed to 94% fucosylation detected in the control conditions on Day 12 without the addition of ThioFuc. The afucosylation level was at 5% for both conditions (Figure 3e). Production of mAb3, mAb4, and the fusion protein in presence of ThioFuc resulted in different thiofucosylation and afucosylation levels compared to the respective control. For clone 3 expressing mAb3, 8% core‐fucosylation, 32% thiofucosylation, and 60% afucosylation were observed on Day 12 in presence of ThioFuc compared to 77% fucosylation and 20% afucosylation in the control (Figure 3f). ThioFuc treatment of clone 4 producing mAb4 resulted in 21% fucosylation, 43% thiofucosylation, and 34% afucosylation on Day 12 compared to 90% fucosylation and 8% afucosylation in the control (Figure 3g). Fusion protein expressed by clone 5 in presence of ThioFuc resulted in 9% fucosylated, 39% thiofucosylated, and 52% afucosylated glycans compared to 71% fucosylated and 27% afucosylated glycans on Day 12 for the control condition (Figure 3h).

Overall, different levels of incorporated ThioFuc were detected on Day 12 for mAb2, mAb3, mAb4, and the fusion protein with about 69%, 32%, 43%, and 39%, respectively. Besides clone 2 (mAb2) having no effect on the afucosylation level, afucosylation was increased for mAb3, mAb4, and the fusion protein by about 39%, 26%, and 24%. Data suggest that ThioFuc might act as competitive substrate to l‐fucose and afucosylation depends on enzyme activity and substrate affinity in each cell line.

### 3.4 Impact of ThioFuc modification on effector functions

**Figure 4:**
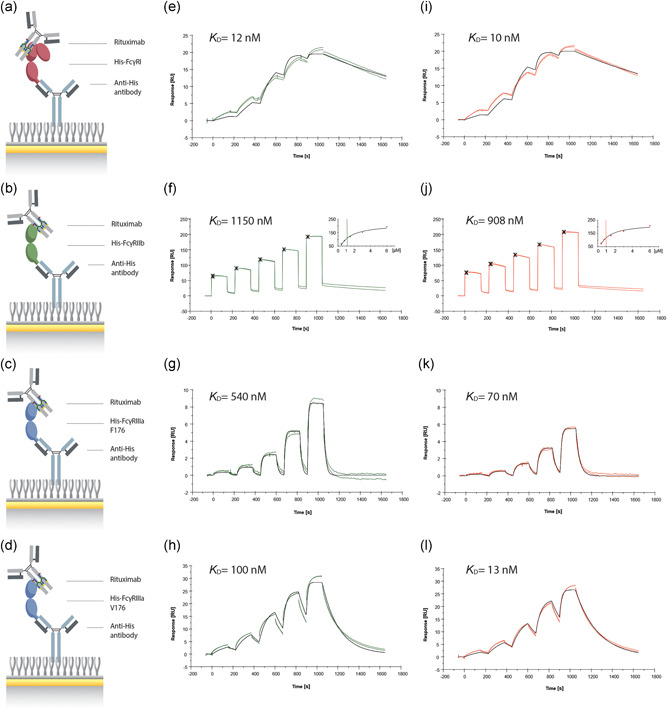
Characterization of a modified rituximab binding to FcγR. A schematic representation of the anti‐His antibody‐mediated capture of (a) FcγRI, (b) FcγRIIb, (c) FcγRIIIa F176, and (d) FcγRIIIa V176 to investigate the binding of each receptor to (e–h) rituximab (8.5% afucosylation) and (i–l) modified rituximab (46% afucosylation and 48% thiofucosylation) using SPR. The Fcγ receptor capture levels were 12 RU for FcγRI, 404 RU for FcγRIIb, 8 RU for FcγRIIIa F176, and 16 RU for FcγRIIIa V176. SPR sensorgrams corresponding to the interactions were obtained via sequential injections for 150 s using single‐cycle kinetics with a final dissociation time of 600 s (*n* = 2). Depending on the Fcγ receptor, antibody concentrations in the range of 7.8–2000 nM for FcγRI and 375–6000 nM for FcγRIIb were injected for both rituximab samples. For the polymorphic variants, F176 and V176 of the FcγRIIIa, concentration ranges of 14.6–1185 nM for rituximab and 1.6–132 nM for the modified rituximab were used. Experimental data are displayed as colored lines, whereas fitted curves after applying the 1:1 binding model are illustrated as black lines. For FcγRIIb binding (f, j), the steady‐state model was applied showing the report points (15 s after injection) as crosses and the resulting steady‐state response is shown in the inset figures with a vertical line indicating the calculated *K*_D_ value. SPR, surface plasmon resonance [Color figure can be viewed at wileyonlinelibrary.com]

Glycosylation of antibodies largely affects their effector functions by interacting with FcγR. The absence of core‐fucosylation is known to enhance ADCC, by increasing the affinity to the FcγRIIIa (Mizushima et al., 2011). As ThioFuc treatment altered the fucosylation pattern, the present study explored the binding of rituximab (8.5% afucosylation) compared to modified rituximab with 46% afucosylation and 48% thiofucosylation to four different FcγR including FcγRIIIa using SPR measurements (Figure 4a–d). The binding was evaluated by the *K*_D_ value via a steady‐state model for quick reactions and otherwise via 1:1 binding model, even though it is known that multiple antibody variants differing in their glycosylation profile can lead to a more complex biological interaction.

High affinity to FcγRI was observed for both rituximab variants, with *K*_D_ values of about 12 nM and 10 nM for the control and modified rituximab, respectively (Figure 4e,i). Low affinity to FcγRIIb was observed for the control and modified rituximab, with *K*_D_ values of 1150 nM and 908 nM, respectively (Figure 4f,j). Thus, thiofucosylation did not change the binding affinity to FcγRI and FcγRIIb, which is in line with previous reports showing no impact of fucosylation on the binding (Bruggeman et al., 2017; Nimmerjahn & Ravetch, 2008).

Furthermore, both variants of the FcγRIIIa were investigated, since the polymorphism at amino acid 176 with either V or F is known to have a strong influence on the binding kinetics (Koene et al., 1997). The binding of rituximab to the low‐affinity variant FcγRIIIa F176 resulted in *K*_D_ values of 540 nM and 70 nM for the control and modified rituximab, respectively (Figure 4g,k). For binding to FcγRIIIa V176, *K*_D_ values of 100 nM for the control and 13 nM for the modified rituximab were observed (Figure 4h,l). In both cases, enhanced binding was mainly attributed to faster association and to some extent slower dissociation compared to the control, for example, association constant (*k*_a_) to FcγRIIIa V176 was increased from 0.69 × 10^5^ M^−1^ s^−1^ in the control to 4.37 × 10^5^ M^−1^ s^−1^ through modified rituximab (Table S2). Overall, FcγRIIIa binding was enhanced for both variants with a 7.7‐fold (F176) and 7.7‐fold change (V176) of the modified rituximab compared to the native form suggesting an enhanced ADCC activity.

**Figure 5:**
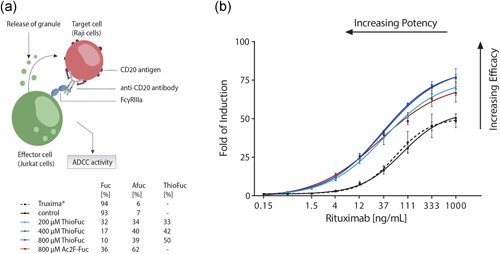
Characterization of the ADCC activity of a modified rituximab. (a) A schematic representation of the ADCC assay from Promega. (b) The ADCC activity of the control condition was compared to ThioFuc and Ac2F‐Fuc treated rituximab samples with different fucosylation profiles (*n* = 2), and to the biosimilar Truxima® (*n* = 5). ADCC, antibody‐dependent cellular cytotoxicity [Color figure can be viewed at wileyonlinelibrary.com]

Finally, this suggested increase in ADCC was further evaluated in a functional assay comparing a thiofucosylated rituximab to the biosimilar Truxima®. At this point, it is important to mention that the goal of the study was to test the effect of ThioFuc supplementation in cell culture media on an industry‐relevant antibody, whose mechanism of action is linked to ADCC and was not to reach biosimilarity. Fed‐batch samples exposed to increasing ThioFuc concentrations of 200–800 µM were analyzed on Day 7 with regard to their ADCC activity. All samples were compared to both Ac2F‐Fuc, serving as the positive control, and the biosimilar Truxima® with a glycosylation profile consisting of 94% fucosylation, 45% terminal GlcNAc, 49% galactosylation, 5% mannosylation, and 1% sialylation (Figure S1c). The ADCC Reporter Bioassay from Promega, which utilizes modified effector cells expressing luciferase upon activation (Figure 5a) was used. All data are visualized as sigmoidal dose–response curves, from which the maximum fold of induction (FI) and half‐maximal effect (EC_50_) were derived—indicating the antibody's efficacy and potency, respectively. The supplementation of ThioFuc showed a higher upper asymptote of the dose–response curves (Figure 5b) for all tested ThioFuc concentrations and the positive control compared to the unmodified rituximab and the biosimilar Truxima®. Upon closer inspection of the maximum FI, the control and Truxima® yielded similar FI values of 55 and 50, respectively. Application of ThioFuc led to an overall increase in the maximum FI and thus efficacy in a dose‐dependent manner, leading to 76, 82, and 83 for 200, 400, and 800 µM ThioFuc, respectively, and 73 FI was detected for the positive control. Furthermore, ThioFuc treatment induced a significant leftward shift of the dose–response curves relative to the control condition and Truxima®. More precisely, the control conditions achieved EC_50_ values of 87 and 63 ng/ml, while the positive control reduced EC_50_ value to 31 ng/ml and all tested doses of 200, 400, and 800 µM ThioFuc reduced the EC_50_ value to 38, 34, and 37 ng/mL—thereby resulting in a major improvement in potency. Overall, generated data demonstrated a 1.5‐fold enhanced efficacy and 2.6‐fold improved potency of rituximab samples produced in presence of ThioFuc. Therefore, ThioFuc proved to be an efficient fucose analogue in boosting the ADCC activity as clinically relevant effector function.

## 4 DISCUSSION

Fucosylation is key for ADCC activity of IgG, which can be modulated by various fucose analogues by inhibition of FUT8 in combination with a substrate feedback inhibition (Allen et al., 2014, 2016; Kizuka et al., 2017; McKenzie et al., 2018; Okeley, Toki, et al., 2013; Rillahan et al., 2012; Zhou et al., 2017; Zimmermann et al., 2019). As the commercial use of these fucose analogues is restricted by patents, our study aimed at developing other derivatives able to increase ADCC. Dose‐response experiments were performed with ThioFuc as a cell culture feed supplement for assessing its ability to reduce core‐fucosylation. The analysis of the glycosylation pattern of modified rituximab by UPLC‐MS revealed a remarkable reduction of core‐fucosylated glycans, with the degree of change becoming more pronounced in a dose‐ and time‐dependent manner. Treatment with ThioFuc reduced core‐fucosylation to a minimum level of 5%, while a concurrent increase in the afucosylation level up to 48% was observed. Further analysis of the glycan data and fragmentation pattern demonstrated the incorporation of ThioFuc to a high level up to 47% in place of core‐fucosylation. These data indicate that ThioFuc is recognized and accepted as donor substrate for catalyzing enzymes in the salvage pathway resulting in guanosine diphosphate ThioFuc (GDP‐ThioFuc) and finally core‐thiofucosylation. Tsuruta et al. (2003) demonstrated the capability of milk α(1,3)‐FUT in utilizing GDP‐ThioFuc as donor substrate, forming a ThioFuc containing trisaccharide thus supporting our observation of GDP‐ThioFuc used as a donor substrate. Since all FUT family members rely on the same substrate (Rillahan et al., 2012), data suggest that, within CHO cells, GDP‐ThioFuc is utilized by FUT8, which is responsible for the fucose attachment via an α(1,6)‐linkage to the innermost GlcNAc residue within the core‐region of the *N*‐glycan.

Based on the extensive CHO cell line screening testing the impact of ThioFuc on other therapeutic proteins, data further demonstrated considerable differences in the thiofucosylation and afucosylation level among each other—strongly suggesting that metabolization of ThioFuc depends on the producing clone, with the extent of incorporation depending on a multitude of factors such as the enzymatic activity, the substrate affinity, and specificity in each cell line. Assuming that ThioFuc is tolerated as a substrate by all enzymes involved in the salvage pathway, its mechanism of action might rely on the competition between thiofucosylation and fucosylation, thereby reducing the level of the latter. Keeping that in mind, accordingly high GDP‐ThioFuc concentration is likely to be expected in the intracellular space. Its accumulation in turn might generate feedback inhibition, resulting in the depletion of the native GDP‐fucose pool and further reduction of core‐fucosylation (Okeley, Alley, et al., 2013; Zandberg et al., 2012). However, it still remains unclear whether reduced core‐fucosylation is based on reduced GDP‐fucose or higher GDP‐ThioFuc levels. Thus, the intracellular nucleotide sugars must be investigated in the future to permit further substantiated conclusions.

As this study clearly confirmed the efficient glycosylation and hence fucosylation modulation by ThioFuc, antibody binding across a panel of four different FcγR was investigated to identify changes in the binding behavior upon ThioFuc treatment and, thus, a possible modulation of the respective effector functions. A side‐by‐side comparison of monitored binding affinities of rituximab to all tested FcγR unveiled the highest affinity for FcγRI and lower affinities for FcγRIIIa V176, FcγRIIIa F176, and finally FcγRIIb, whereby the order of descending affinity was also described elsewhere (Bruhns et al., 2009). However, ThioFuc treatment only appeared to affect the antibody binding to both variants of the FcγRIIIa. The observed enhancement in antibody‐FcγRIIIa binding strength was mainly attributed to faster association rate constants and, to some extent, to slower dissociation rate constants. These results are in agreement with those reported in the literature, where changes in *k*_a_ and *k*_d_ values due to reduced core‐fucosylation have been demonstrated (Ferrara et al., 2006; Hayes et al., 2014; Okazaki et al., 2004). Okazaki et al. (2004) concluded that the increase in association rate constants might be explained by the fact that the binding activation energy, which must be overcome for conformational rearrangement upon complex formation, is lowered with the reduction of the fucosylation proportion. Furthermore, slower dissociation of modified antibodies from FcγRIIIa was observed, also contributing to increased affinity. Ferrara et al. (2006) and Hayes et al. (2014) reported similar findings—with the slower dissociation being possibly attributable to newly formed or enhanced binding interactions, thereby leading to a conformational change and stabilization of the antibody‐FcγR complex over the analyzed time period. Another explanation might be the time‐dependent accumulation of thiofucosylated/afucosylated antibodies on the sensor chip surface, assuming that these antibody species are characterized by slower dissociation from the receptor (Hayes et al., 2014).

Based on enhanced antibody‐FcγRIIIa binding, the impact of ThioFuc on the ADCC activity was further evaluated by an in vitro ADCC assay. Consistent with several reviews describing the greater efficiency of low‐fucosylated antibodies in inducing ADCC compared to their fucosylated counterpart (Jennewein & Alter, 2017; Pereira et al., 2018; Satoh et al., 2006), modified rituximab did exert a change in the efficacy and potency, both of which account for a comparatively higher ADCC activity than the native variants. According to the results of Chung et al., the increase in the efficacy appears to be attributed to the increased afucosylation proportion in the sample. This assumption is supported by their experiment, in which they have generated a multitude of antibody samples with varying afucosylation levels from 0% to 100%. The dose–response curves of the respective samples were characterized by a substantial left and upward shift, indicating an increasing potency and efficacy, which correlated well with the afucosylation level (Chung et al., 2014). In conclusion, these data indicate that the enhanced FcγRIIIa‐mediated binding is the chief driver of the observed effects on efficacy. Therefore, it can be concluded that human plasma IgG, as part of the ADCC assay, is displacing fucosylated antibodies more easily compared to the ThioFuc modified antibodies, exhibiting a much higher binding strength and thus remain bound to the receptor. Such modified antibodies are thought to activate effector cells and downstream signaling pathways more efficiently, whereby this activation likely leads to increased efficacy.

Overall, ThioFuc was proven to be an efficient and potent fucosylation modulator, able to enhance the antibody binding strength to the FcγRIIIa and consequently ADCC activity reflecting the antibody's cytotoxic efficacy. A direct comparison of ThioFuc and Ac2F‐Fuc revealed differences in their efficiency in modulating fucosylation across multiple CHO cell lines. Thus, new production processes might screen several fucose analogues to determine the most suitable treatment leading to a reduced core‐fucosylation. Despite the promising findings of this study, it remains unclear whether these observations were attributed to thiofucosylation or solely increased afucosylation. Two strategies might be tested to assess the impact of thiofucosylation. First, a mixture with the same percentage of afucosylation and additional thiofucosylation might be compared, or second, separation and isolation of thiofucosylated glycans from a mixture of heterogeneous fucosylated and afucosylated glycans may allow an unbiased interpretation of the impact of thiofucosylation. As such modified antibodies with superior biological properties might be used as therapeutic drugs, future studies are essential to gain more knowledge and determine any effects occurring in living organisms.

## CONFLICT OF INTERESTS

The authors declare that they have no competing financial interests. All authors are employees of Merck KGaA except Harald Kolmar, who is an employee of the Technische Universität Darmstadt.

## AUTHOR CONTRIBUTIONS

Martina Zimmermann and Aline Zimmer conceived the study. Martina Zimmermann is responsible for experimental design. Martina Zimmermann, Melanie Nguyen, and Christian M. Schultheiss conducted the experiments. Martina Zimmermann, Melanie Nguyen, Harald Kolmar, and Aline Zimmer analyzed and interpreted the data. Martina Zimmermann drafted the manuscript. Martina Zimmermann and Melanie Nguyen prepared the figures. Melanie Nguyen, Christian M. Schultheiss, Harald Kolmar, and Aline Zimmer revised the manuscript. All authors read and approved the final manuscript.

## Supporting information

Supporting information.

## Data Availability

The data that supports the findings of this study are available in the supplementary material of this article.
