# Supplementary material for: Use of 5‐Thio‐L‐Fucose to modulate binding affinity of therapeutic proteins
Source: Biotechnol Bioeng. 2021 Feb 19;118(5):1818–31. doi: 10.1002/bit.27695 (PMC8248388; doi:10.1002/bit.27695)
Supplement: Supplementary file 1 — Supporting information. [file BIT-118-1818-s001.docx]

# Supplementary data

**Figure S1. ThioFuc dose response during fed-batch experiments using a rituximab (mAb1) producing clone.** Suspension CHO cells were seeded at 3×105 cells/mL, incubated at 37°C, 5% CO2, 80% humidity and agitated at 320 rpm. Feed with increasing ThioFuc or Ac2F-Fuc concentrations was added on day 3, 5, 7, 10 and 12 (5%; v/v). Integral over time of (A) viability and (B) IgG visualizes the cell performance and (C) glycosylation profile on day 12 (n=2).

Table S1. Glycan structures detected on day 12 upon treatment with ThioFuc and Ac2F-Fuc compared to the control condition. Using rituximab produced in presence of both fucose analogues, the respective N-glycans were released and labelled. All presented N-glycans were identified by UPLC-MS analysis according to their specific retention time and mass to charge ratio. The respective glycans were quantified by calculating the ratio of the corresponding peak to the total peak area. The glycan data are shown as mean values.

Table S2. Kinetic data.


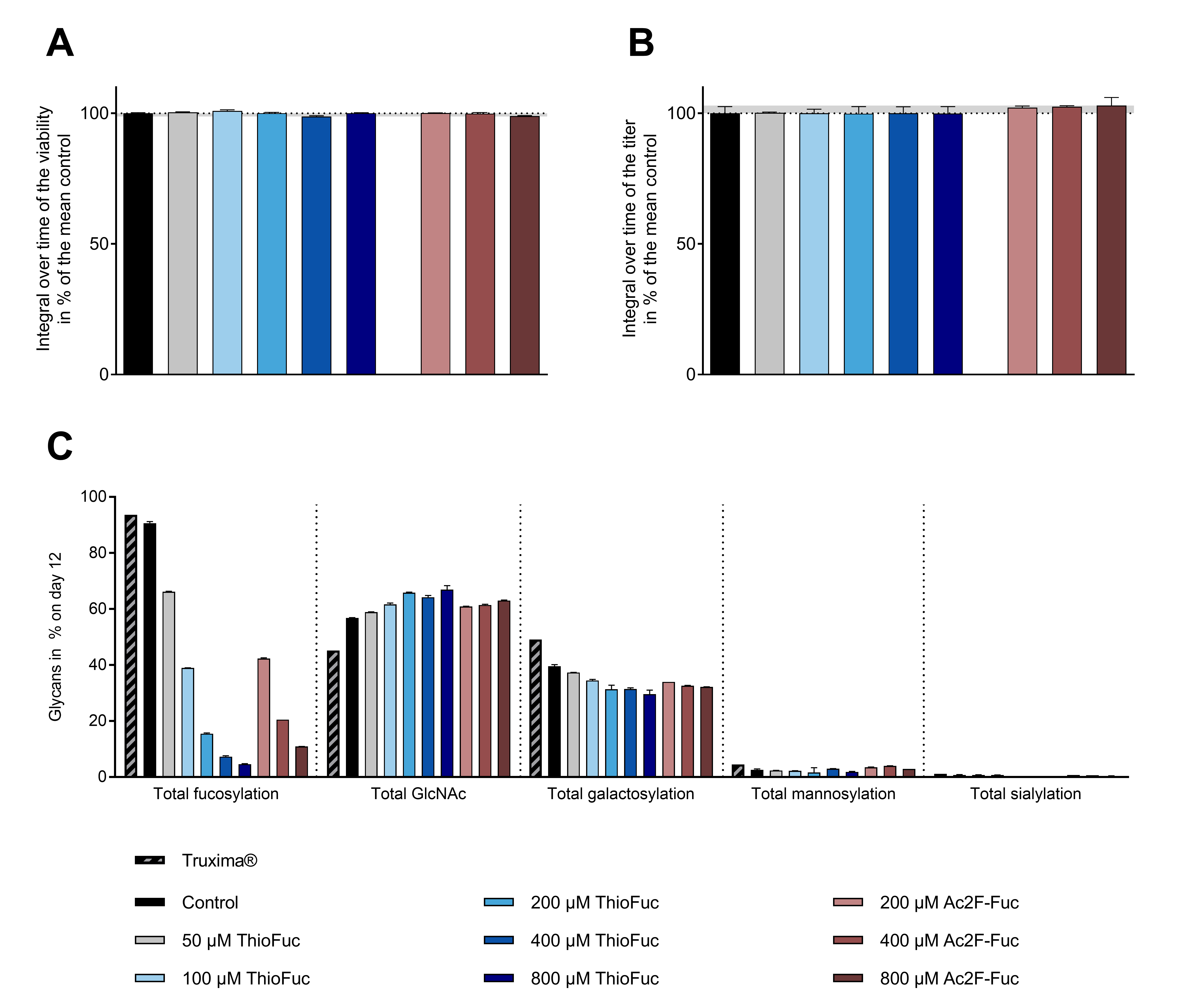


**Figure S1**

**Table S1**

| Name | Structure | Control | 50 µM  ThioFuc | 100 µM  ThioFuc | 200 µM  ThioFuc | 400 µM  ThioFuc | 800 µM  ThioFuc | 800 µM  Ac2F-Fuc |
| --- | --- | --- | --- | --- | --- | --- | --- | --- |
|  |  | (n=4) | (n=2) | (n=2) | (n=2) | (n=2) | (n=2) | (n=2) |
| G0F | 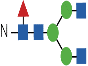 | 52.2 | 38.0 | 22.2 | 9.3 | 4.2 | 2.9 | 6.1 |
| G1F | 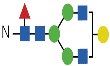 | 32.7 | 24.0 | 14.3 | 5.5 | 2.9 | 1.7 | 4.1 |
| G2F | 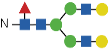 | 4.2 | 3.0 | 1.8 | 0.7 | 0.3 | – | 0.5 |
| G0F-N | 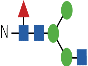 | 0.9 | 0.6 | 0.3 | – | – | – | – |
| G0ThioF | 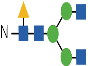 | – | 9.4 | 19.4 | 27.5 | 29.6 | 32.9 | – |
| G1ThioF | 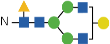 | – | 3.8 | 8.2 | 11.7 | 11.9 | 12.9 | – |
| G2ThioF | 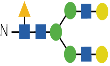 | – | 0.7 | 1.1 | 1.1 | 1.5 | 1.2 | – |
| G0 | 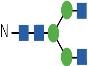 | 3.5 | 10.7 | 19.4 | 29.0 | 30.2 | 31.0 | 56.7 |
| G1 | 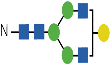 | 1.7 | 4.6 | 7.7 | 11.4 | 12.9 | 12.6 | 24.1 |
| G2 | 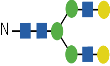 | 0.3 | 0.4 | 0.7 | 0.9 | 1.4 | 0.9 | 2.4 |
| Man5 | 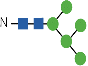 | 1.7 | 1.5 | 1.5 | 1.7 | 2.7 | 1.7 | 2.8 |

Table S2

| Receptor | Sample* | *k*_a_  (×10^5^ M^-1^ s^-1^) | *k*_d_  (×10^-3^ s^-1^) | *K*_D_ ± SEM  (nM) | Fold change† |
| --- | --- | --- | --- | --- | --- |
| FcγRI | rituximab | 0.49 | 0.67 | 14 ± 2.3 | – |
|  | modified rituximab | 0.60 | 0.68 | 11.3 ± 1.5 | 1.2 |
| FcγRIIb | rituximab | – | – | 1140 ± 17 | – |
|  | modified rituximab | – | – | 893 ± 43 | 1.3 |
| FcγRIIIa F176 | rituximab | 1.1 | 57.4 | 540 | – |
|  | modified rituximab | 4.6 | 33.4 | 72 ± 2 | 7.5 |
| FcγRIIIa V176 | rituximab | 0.70 | 7.0 | 100 ± 5 | – |
|  | modified rituximab | 4.37 | 5.7 | 13 ± 0.4 | 7.7 |

* rituximab (8.5% afucosylation), modified rituximab (46% afucosylation and 48% thiofucosylation)

† Fold change refers to the average *K_D_* values of rituximab and modified rituximab samples
